# The effect of aerobic and resistance training in patients with type 2 diabetes on vitamin D (DIAVITEX): a study protocol

**DOI:** 10.3389/fpubh.2025.1674293

**Published:** 2026-01-05

**Authors:** Elnaz Dardashtipour, Sílvia Canivell, Mohammad Ali Azarbayjani, Andrea Fuente-Vidal, Aina Surroca, Pilar Gascón, Concepció Mestres, Alícia Antón, Maria José Peña-Mateo, Elena Carrillo-Alvarez, Anna Maria Canudas, Myriam Guerra-Balic, Joel Montané

**Affiliations:** 1 Facultat de Psicologia, Ciències de l'Educació i de l'Esport Blanquerna, Ramon Llull University, Barcelona, Spain; 2 Centre d’Atenció Primària Adrià, Gerència Territorial de Barcelona, Institut Català de la Salut, Barcelona, Spain; 3 Department of Exercise Physiology, CT.C., Islamic Azad University, Tehran, Iran; 4 Facultat de Ciències de la Salut, Blanquerna, Ramon Llull University, Barcelona, Spain; 5 Centre d’Atenció Primària Sant Rafael, Gerència Territorial de Barcelona, Institut Català de la Salut, Barcelona, Spain; 6 Department of Pharmacology, Toxicology and Therapeutic Chemistry, Faculty of Pharmacy and Food Sciences, Institute of Neurosciences, University of Barcelona, Barcelona, Spain

**Keywords:** resistance training, vitamin D, type 2 diabetes, insulin resistance, clinical trial

## Abstract

**Introduction:**

Aerobic and resistance training can effectively improve clinical management in people with type 2 diabetes (T2D). Low vitamin D (VitD) levels are associated with T2D risk and metabolic disturbances, and may help reduce this risk, particularly in individuals with low VitD levels. In this line, many individuals with T2D, who may also be older adults or have osteoporosis, regularly include VitD treatment in their healthcare routines. Although the impact of exercise has been extensively studied, its effect on diabetic patients taking VitD remains limited. The aim of this study is to investigate the effect of aerobic and resistance training on clinical parameters in patients with T2D already taking VitD.

**Methods:**

The DIAVITEX study is a randomized controlled superiority trial, with four parallel arms, including 80 individuals with T2D. Patients will be selected at the Primary Care Centers and stratified according to their pre-existing VitD treatment. Participants will subsequently be randomized to the exercise intervention or control as follows: Group 1, Exercise + VitD users (*n* = 20); Group 2, Exercise + VitD non-users (*n* = 20); Group 3, VitD only (no exercise) (*n* = 20); and Group 4, Control (No VitD & No Exercise) (*n* = 20). In this study, a sarcoplasm-stimulating training program will be carried out online, three sessions per week for a total of 16 weeks. Before and after the physical activity subjects will perform fitness and blood tests. Nutritional education programs will be provided to normalize their diets for study consistency. The primary endpoint of the trial is the change in HOMA-IR index from baseline to week 16. Secondary endpoints include changes in HbA1c, lipid profile, body composition, and inflammatory biomarkers.

**Discussion:**

Expected improvements in insulin resistance, glycated hemoglobin, lipid profile, and inflammatory markers are anticipated following a 16-week regimen of exercise in patients with T2D on VitD.

**Clinical trial registration:**

The study was registered on September 21, 2024, with the identifier number NCT06081387, https://clinicaltrials.gov/study/NCT06081387.

## 1 Introduction

Type 2 diabetes (T2D) is a chronic disease characterized by insulin resistance and impaired pancreatic insulin production. The rapid growth of this epidemic reflects socioeconomic trends, including, among others, low levels of physical activity (PA) (1). In this regard, obesity and lack of regular exercise or a sedentary lifestyle are major causes of chronic disease and global mortality, in which PA plays a very significant role (2).

Low cardiorespiratory fitness (CRF) is a well-known risk factor and an important predictor of mortality for chronic diseases including T2D and obesity (3, 4). Aerobic and resistance training is the most effective way to increase CRF, and people with T2D clearly respond to training with this adaptation (5). Aerobic exercise interventions have shown to improve glycemic parameters, VO2 max and cardiac output in T2D, which was associated with a significant reduction in the risk of cardiovascular and mortality (5–7). Other studies have shown improvements of 10 to 15% in strength, bone mineral density, blood pressure, lipid profiles, cardiovascular health, insulin sensitivity, abdominal fat and muscle mass (6). Additionally, due to the increased prevalence of T2D with age and the age-related loss of muscle mass, known as sarcopenia, resistance training may have additional health benefits for older adults (8). As a result, PA has been established as an important therapy in the prevention, management, and treatment of T2D and its associated complications (9, 10).

Vitamin D (VitD) is a fat-soluble vitamin innately present in some foods. VitD promotes intestinal calcium absorption, maintains adequate serum calcium and phosphate concentrations to afford normal bone mineralization, and prevents hypocalcemic tetany (11). Together with calcium, VitD also helps protect older adults from osteoporosis. VitD has other physiological functions, including reducing inflammation and modulating processes such as cell growth, neuromuscular and immune function, and glucose metabolism (11). Thus, it is widely acknowledged that a significant portion of individuals with T2D, which may also present with advanced age, or osteoporosis, routinely integrate a VitD treatment into their healthcare routines (12). Low blood levels of VitD have emerged as a risk factor for TD2; it is often associated with metabolic alterations, such as an increased risk of glucose intolerance (13). Further, obesity is inversely correlated with VitD status, which can be attributed to the sequestration of soluble vitamins by adipose tissue (14). Furthermore, it has been found that low VitD levels are associated with insulin resistance and insulin secretion disorders leading to the development of TD2 (15). Therefore, VitD may play a functional role in glucose tolerance through its effects on insulin secretion and insulin sensitivity (16). In this line, several studies have hypothesized that VitD supplementation is a potential intervention to reduce the risk of T2D. In addition, other studies have shown that VitD is beneficial for people with pre-diabetes (17). However, VitD supplementation alone as an intervention cannot improve insulin sensitivity or glycemic control in an overweight population with previously normal VitD levels (18). Likewise, VitD supplementation also fails to improve glucose tolerance or pancreatic beta-cell function in patients with pre-diabetes.

Few studies evaluated the effects of VitD and PA in patients with T2D. Kim et al. showed that the combination had positive effects on abdominal fat and blood lipid profiles, but the metabolic characterization of these patients was limited, and results were inconclusive (19). Dadrass et al. showed that VitD supplementation, in addition to resistance training, had positive effects on some inflammatory markers (interleukin 6, tumor necrosis factor *α* and C-reactive protein) in men with T2D and VitD insufficiency but they did not analyze any non-inflammatory metabolic parameters (20). In conclusion, although some studies link VitD and aerobic or endurance exercise to improved T2D outcomes, the analyses are scattered and inconsistent, with incomplete metabolic characterization, low numbers of patients, and generally unreliable conclusions.

To overcome these limitations, the DIAVITEX study employs a randomized factorial design with a larger sample size and stratification by VitD treatment status, allowing the independent and combined effects of exercise and VitD to be assessed. It also incorporates a comprehensive metabolic characterization (including insulin resistance, glycemic, lipid, and inflammatory biomarkers) and a 16-week structured, supervised exercise program to ensure adherence and reproducibility. Involving pharmacists and primary care professionals further enhances safety and translational applicability in real-world clinical settings.

The DIAVITEX study aims to identify the effect of aerobic and resistance training in T2D patients on a VitD regime and to compare them with patients not exercising or not on VitD therapy, in order to provide evidence for optimizing disease management. The specific objectives are to evaluate the effect of aerobic and resistance training, with or without VitD treatment, on:

1. Insulin resistance (primary endpoint: HOMA-IR).
2. Glycemic, lipid, inflammatory and renal biomarkers.
3. Anthropometric measures.
4. Adherence to the Mediterranean diet.
5. Psychological well-being among patients with T2D before and after the intervention.
6. The role of the pharmacist in patient follow-up including medication monitoring, intervention support, and safety oversight during the trial (exploratory process outcome).

We hypothesize that the combination of aerobic and resistance training with ongoing VitD treatment will lead to greater improvements in insulin resistance (primary endpoint: HOMA-IR) compared with exercise alone, VitD alone, or control (no intervention). Secondary hypotheses are that exercise, with or without VitD, will also improve glycemic control, lipid profile, body composition, inflammatory markers, diet adherence, and psychological well-being.

## 2 Methods and analysis

### 2.1 Study design

The DIAVITEX study is a randomized controlled clinical trial involving medicinal products, designed in accordance with Real Decreto 957/2020 of November 3rd, which regulates observational studies with human-use medicinal products. The trial framework is superiority, designed to test whether the combination of aerobic and resistance training with ongoing VitD treatment is superior to either intervention alone or control in improving insulin resistance.

### 2.2 Participants

The study will include a total of 80 individuals of both sexes (female, male) with a diagnosis of T2D. Among them, 40 individuals will be required to be on a VitD treatment for at least, 6 months, for reasons recommended by the ECAP working group (including osteoporosis, osteopenia, and VitD deficiency), and the other 40 will not be receiving VitD supplementation.

Subjects will be recruited during usual medical care in a convenience-based by the collaborating physician from the Primary Care Center (CAP) Sant Rafael in Barcelona, Spain.

During the initial visit with the physician at CAP Sant Rafael, participants will be provided with the Patient Information Sheet and the Informed Consent and subsequently will be asked to provide their preferred contact information (phone number or email). This information will be used by the principal investigators from Blanquerna to reach out explaining the study with detailed instructions (explaining the patient information sheet and address any questions they may have). After receiving the information and clarifying any potential concerns, participants will be asked to sign the informed consent.

After enrollment and stratification by VitD treatment status, participants will be randomized within each stratum to exercise intervention or to no exercise. An independent investigator, not involved in recruitment or outcome assessment, will generate the randomization sequence using a computer-based block randomization method within VitD strata. Allocation will be implemented via a secure, web-based central randomization system accessible only after completion of baseline assessments and confirmation of eligibility. This ensures that the four groups (see Table 1) are balanced and clearly defined as: Group 1, Exercise + VitD users; Group 2, Exercise + VitD non-users; Group 3, VitD only (no exercise); and Group 4, Control (no VitD & no exercise).

**Table 1:** Group allocation of participants with T2D.

Group	VitD treatment status	Exercise intervention	*n*
1	On VitD ≥ 6 months	Exercise program	20
2	No Vit D treatment	Exercise program	20
3	On VitD ≥ 6 months	No exercise	20
4 (Con)	No Vit D treatment	No exercise	20

The sample size for this study was determined using the GRANMO program with the model of observed proportions with respect to a reference. For the calculation, it has been taken into account that the insulin resistance is the key to calculating the effects of aerobic and resistance training on clinical parameters in patients with T2D. The calculation has been made accepting an 𝜶 risk of 0.05 and a beta risk of less than 0.2 in a two-sided contrast. Based on preliminary data, there is a difference of HOMA-IR of -0.14% after 12 weeks of intervention (21). Although modest, this effect size has been reported as clinically relevant in previous clinical trials (22–24). It is assumed that the proportion in the reference group is 0.0139 (5.3 million people in Spain) (25). The percentage of necessary replacements has been predicted to be 20%. The present study is designed as an exploratory factorial randomized clinical trial; while not powered for a definitive test of interaction effects, the chosen sample size allows us to detect main effects with adequate power while providing preliminary evidence of potential interactions.

### 2.3 Inclusion/exclusion criteria

Inclusion criteria for the subjects of this study are detailed below:

- Adults (females or males) older than 18 years old with a diagnosis of T2D in the clinic database.
- Patients who have been taking the combination therapy of metformin + sodium-glucose transport protein 2 inhibitors (iSGLT2), as recommended by the redGDPS 2023, with stable medication for the past 6 months.
- Diabetic patients taking prescribed VitD treatment for at least 6 months (intervention group) and diabetic patients not taking prescribed VitD (control group).
- Patients who signed the informed consent.
- Patients capable of performing mild to moderate physical activity (to walk steadily and independently for at least 6 min).

Exclusion criteria of subjects are summarized below:

- Patients taking other medication different than metformin + iSGLT2 (including combinations and insulin).
- Patients with severe or uncontrolled chronic diseases (e.g., cardiovascular, hepatic, renal, or musculoskeletal disorders) that could interfere with exercise performance, metabolic outcomes, or overall safety.
- Patients taking polyvitaminic supplementation at the inclusion for at least, 1 month before the intervention.
- Female subjects who are pregnant.
- Patients who did not sign the informed consent.

### 2.4 Endpoints

#### 2.4.1 Primary endpoint

The primary endpoint of the DIAVITEX trial is the change in insulin resistance, measured by the HOMA-IR index, from baseline to week 16.

#### 2.4.2 Secondary endpoints

Secondary endpoints include:

- Changes in glycemic parameters (HbA1c, fasting glucose, fasting insulin).
- Changes in lipid profile (total cholesterol, HDL, LDL, triglycerides).
- Changes in inflammatory markers (C-reactive protein, interleukin-6, TNF-*α*).
- Changes in anthropometric parameters (weight, BMI, waist circumference, skinfold thickness).
- Changes in psychological well-being and adherence to the Mediterranean diet.
- Safety outcomes, including incidence of hypo-and hyperglycemia and any adverse events related to the intervention.

### 2.5 Variables

#### 2.5.1 Anthropometric measurements

After measuring height and weight, body mass index will be calculated with the following formula:


$$\mathrm{BMI}=\text{weight}(\mathrm{kg})/\text{height}(m^{2})$$

To calculate the percentage of body fat, the subcutaneous fat of the subjects will be measured using a skin thickness gauge in three areas of the abdomen, upper extremities and arms. All skinfold measurements will be performed on the right side of the body in 3 turns with an interval of 20 s to return to the original position. An average of 3 measurements will be recorded and the three-point formula of the American College of Sports Medicine will be used to calculate the percentage of body fat:


$$\begin{array}{c} \mathrm{Fat}\;\text{percent}={\left(0.39278\right)}^{*}(\text{total of three parts})\\ -0.00105^{*}(\text{total of three parts})2\\ +[0.15772^{*}(\mathrm{Age})]-5.18845 \end{array}$$

Anthropometric parameters will be taken by the standardized method of the International Society for the Advancement of Kinanthropometry (ISAK).

#### 2.5.2 Food consumption and adherence to the Mediterranean diet

Nutritional questionnaires will be provided in person during the first visit at the participating pharmacies, right after recruitment. Adherence to the Mediterranean dietary pattern will be determined using the Mediterranean Diet Adherence Screener (MEDAS) developed in the PREDIMED study. MEDAS is a questionnaire consisting of 14 questions. Values of 0 or 1 are assigned to each item, the maximum adherence to the Mediterranean Diet is indicated by a 14-point score (26). A validated food frequency questionnaire (FFQ) with 137 items plus specific questions for patterns of alcohol consumption and vitamin/minerals supplements will also be administered (adapted from the Willett questionnaire and validated in Spain) (27).

The inclusion of these dietary assessments aims to provide valuable insights into the eating habits of the selected population before the study. Based on the participant’s diet, during the study we will reinforce the recommendations of the Spanish Society for Diabetes. Nutritional education programs based on the previously mentioned recommendations will be provided to participants, where adaptations can be designed to participant particular needs (age, gender, activity, DM treatment, etc.) (28). Moreover, the gathered data will also serve to normalize the overall diet of all participants during the study.

In addition to the questionnaires, dietary intake and adherence will be briefly reviewed during the follow-up via monthly telephone interviews and pharmacy visits to check consistency of responses and reinforce adherence. Participants may also keep short dietary notes or logs if feasible.

#### 2.5.3 Activity degree and psychological well-being

Activity degree and psychological well-being questionnaires will be provided during the first visit at the participating pharmacies, right after recruitment. To evaluate the degree of activity, the questionnaire validated in Catalan, Qüestionari Internacional d’Activitat Física (29), will be administered to measure the physical activity, which through an interview assessing different items will allow to obtain the usual physical activity, being able to classify individuals in sedentary, moderate sedentary or active lifestyles and will serve to evaluate overall physical activity degree for further comparisons and analysis.

The psychological subjective well-being will be measured through the Psychological Well-being Index (IBP; Índex de Benestar Psicològic), which refers to happiness or well-being. Therefore, greater subjective perception of well-being will give higher scores (30). This comprehensive assessment will be conducted both before and after the intervention, allowing for a comparative analysis of changes in psychological well-being over the course of the study.

#### 2.5.4 Blood sampling and analysis

A total of 2 blood samples will be obtained during the study. The analyses will be conducted at the following times:

- After the first visit, at baseline, and before the intervention (complete blood analysis, including VitD).
- At the end of study, at month 4, after the exercise intervention (complete blood analysis, including VitD).

All subjects will go in the morning at their respective Primary Care Centers after 12 h of fasting. Blood sampling will be performed at the corresponding CAP. Blood samples will be analyzed by the Servei de Bioquímica Clínica (Hospital Universitari Vall d’Hebron).

The blood parameters to be determined are the following:

- Vit D (cholecalciferol)
- Insulin
- Glucose
- Glycosylated hemoglobin
- Total Cholesterol
- LDL cholesterol
- HDL cholesterol
- Triglycerides
- Hepatic profile
- Microalbuminuria
- Hemogram
- Renal function
- C reactive protein
- Erythrocyte sedimentation rate
- Ferritin

HOMA-IR index will also be calculated as previously described (31) by using formula (HOMA-IR = fasting glucose in mmol/l*fasting insulin in μU/ml/22.5) to determine insulin resistance. HOMA-B index to quantify the pancreatic beta cell function using formula (HOMA-B360 x fasting insulin (μU/mL)/(fasting glucose (mg/dL)−63)) will be determined.

#### 2.5.5 Pharmacist role

The role of the pharmacist in patient follow-up will be evaluated as an exploratory process endpoint. This includes documenting completion of scheduled follow-up visits, administration of dietary and psychological questionnaires, monitoring of medication adherence, and reporting adverse events.

Pharmacists involved in the study will be licensed professionals, also PhD holders or doctoral candidates, trained by the study team in protocol procedures, participant education, and safety monitoring.

### 2.6 Familiarization with resistance training to measure one maximum repetition (1RM)

During an initial familiarization session, the subjects will be instructed how to properly perform the resistance training program and will receive detailed safety recommendations. The trainer will first demonstrate the movements, after which participants will practice them without weights. Real-time feedback will be provided to ensure correct technique and reduce the risk of injury. Maximum strength or one maximum repetition (1RM) in all movements will not be directly tested, but will be indirectly estimated online using the validated Brzycki equation (32):


1RM=lifted weight(kg)/1.0278−(frequencies×0.0287)

Training intensities during the intervention will be prescribed as a percentage of the estimated 1RM and will be further adjusted according to participants’ perceived exertion and feedback to ensure both safety and adherence.

### 2.7 Sarcoplasm-stimulating training program

A sarcoplasm-stimulating training program will be used as the primary intervention model. This program combines resistance and aerobic components with short rest intervals between sets, designed to increase metabolic stress and enhance both muscular endurance and insulin sensitivity. Such adaptations are particularly relevant in patients with T2D, where resistance training has been shown to improve glycemic control, body composition, and inflammatory status (6, 8, 20). In addition, this training format can be implemented safely in an online, home-based setting without the need for specialized equipment, making it feasible and scalable in real-world primary care contexts.

This is a home training program where sessions are conducted online via corporate Teams from Blanquerna, Universitat Ramon Llull. Participants will not require any additional material, as the physical activity program has been specifically designed to be performed without equipment. Participants will receive in person by the investigator and personal trainer, the link and access codes during their initial visit to the community pharmacy.

The program consists of one familiarization week followed by three progressive 5-week phases (weeks 1–15), each with gradually increasing intensity and reduced rest intervals (see Table 2). In Week 1 after familiarization, participants will perform 4 sets of 8 repetitions at 60% of 1RM, with 20 s of rest between sets. In Week 2, the same format will be maintained with slightly higher intensity (65% 1RM). In Week 3, each movement will be performed in 5 sets of 6 repetitions at 75% of 1RM, with 20 s of rest. In Week 4, the number of sets and repetitions will be maintained but the intensity of training will increase to 80% of 1 RM. In Week 5, considered a tapering phase, participants will perform 5 sets with 4 repetitions at 70% of 1RM, again with 20 s of rest between each set. This 5-week progression will be repeated and advanced through Weeks 6–10 and Weeks 11–15.

**Table 2:** Distribution of the sarcoplasm-stimulating training program (weeks 1–15).

Phase	Week	Sets × repetitions	Rest interval (seconds)	Intensity (% 1RM)
Familiarization	–	Technique practice, no external load	–	–
Phase 1	1	4 × 8 reps	20	60%
	2	4 × 8 reps	20	65%
	3	5 × 6 reps	20	75%
	4	5 × 6 reps	20	80%
	5 (taper)	5 × 4 reps	20	70%
Phase 2	6	4 × 8 reps	20	60%
	7	4 × 8 reps	20	65%
	8	5 × 6 reps	20	75%
	9	5 × 6 reps	20	80%
	10 (taper)	5 × 4 reps	20	70%
Phase 3	11	4 × 8 reps	20	60%
	12	4 × 8 reps	20	65%
	13	5 × 6 reps	20	75%
	14	5 × 6 reps	20	80%
	15 (taper)	5 × 4 reps	20	70%

1RM: maximum repetition.

All exercises are designed as a combination of aerobic and resistance training, following the principle of sarcoplasm stimulation, which emphasizes reduced rest intervals to enhance metabolic stress and muscular adaptation (6, 8, 20).

The total duration of 16 weeks, including one familiarization week, was selected based on previous studies reporting significant metabolic and functional adaptations within 12–16 weeks of combined aerobic and resistance training in patients with T2D (21, 24). This time frame provides sufficient physiological stimulus to detect improvements in insulin resistance, strength, and body composition while maintaining participant adherence in a home-based program.

To enhance adherence and ensure correct execution of the home-based, online exercise program, several strategies will be implemented. First, participants will attend the initial familiarization session, during which individualized feedback will be provided to ensure proper technique and understanding of the training protocol. Second, all exercise sessions will be delivered as life online, supervised in real time by the personal trainer who will monitor attendance and performance. Third, participants will attend monthly follow-up visits at the collaborating pharmacies, where pharmacist (in collaboration with the personal trainer) will review adherence, discuss potential difficulties and provide reinforcement of study instructions. Fourth, monthly telephone interviews will be conducted by the study team to assess adherence, identify any adverse events, and resolve technical issues related to the online delivery of the program. In addition, participants will be asked to complete self-reported exercise logs documenting session completion, perceived effort and any difficulties encountered. These logs will be collected and reviewed monthly by the personal trainer to ensure compliance and to provide tailored feedback when necessary.

Personal trainers involved in the study will hold accredited university degrees in Sport Sciences and will be either PhD holders or currently enrolled in a doctoral program, with prior experience prescribing and supervising resistance and aerobic exercise in clinical populations.

### 2.8 Current medication

During the first visit at the CAP, participating physicians will collect information regarding the current medication of diabetic patients. This information will serve as baseline data and aid in the interpretation of outcomes, adjustment in statistical analyses, and support a more individualized approach to patient care. Additionally, the data can be utilized to identify common treatment patterns, assess adherence to prescribed therapies, and explore the relationship between medication regimens and patient outcomes.

### 2.9 Participant visits and follow-up

A total of 6 visits are expected per each participant. The study includes a first visit at the designated CAP for the explanation of the study, right before the intervention. During this first visit, physicians from CAP Sant Rafael will thoroughly assess participants to confirm their eligibility for the study. During the initial visit, the physicians will provide potential participants with the Patient Information Sheet and the Informed Consent allowing them time to review the documents at home. During this first visit, participants will be asked to provide their preferred contact information (phone number or email), which will be later used to send detailed instructions, the list of participating pharmacies, and to initiate their participation in the study. Blanquerna investigators will explain the study protocol using the patient information sheet and will set a meeting at the community pharmacy. Pharmacies will collaborate solely by providing the space, while all study activities will be conducted by the investigator’s pharmacist from Blanquerna.

Once the patients signed the informed consent, blood samples will be requested by the physicians from CAP Sant Rafael for clinical analysis; this analysis will take place during the second visit at the CAP.

The collaborating community pharmacies from the geographical area of the study can be seen in Table 3.

**Table 3:** Collaborating pharmacies near CAP Sant Rafael.

Pharmacy	Contact information
Perez Sanchez, Ma Teresa	Roig i Solé 5-7, 08035 Barcelona. Tel: 654024765
Rodríguez Poveda, M. Isabel	Vall d’Hebron 130-134, 08035 Barcelona. Tel: 935206870
Hernández Molina, David	Santa Rosalia 82, 08035 Barcelona. Tel: 934291090
Alonso Ramos, Eva	Murtra 18, 08032 Barcelona. Tel: 934294013
Gardella Roca, Neus	Arenys 108, 08035 Barcelona. Tel: 934295714
Calderon Olivas, Stella	Lledoner 2, 08035 Barcelona. Tel: 934175222
Sanroman Garcia, Blanca	Pla de Montbau 7, 08035 Barcelona. Tel: 934287229
Marimon Carvajal, Anna Mª	Av. Elies Pagès 20, 08035 Barcelona. Tel: 934171123
Caballero Perez, Sandra	Santa Rosalia 31, 08035 Barcelona. Tel: 934298889
Gomez Gomez, Ma. Angeles	Naim 1, 08035 Barcelona. Tel: 932111892
Esquerra Amargos, Montserrat	Angel Marquès 7, 08035 Barcelona. Tel: 934283995
Martinez Sanvinsens, Jordi	L’Harmonia 1A, 08035 Barcelona. Tel: 934282899
Pagano Roson, Felix Julio	Juan de Mena 1-3, 08035 Barcelona. Tel: 934282867
Pardo Garcia, Tino	Berenguer de Ste. 56, 08035 Barcelona. Tel: 934282827
Carbo Pallares, Julio Francisco	Arenys 25, 08035 Barcelona. Tel: 932112460
Artiles Leyes, Laura	Idumea 14-16, 08035 Barcelona Tel: 650817408
Yeregui Farran, Jorge	Harmonia 31, 08035 Barcelona Tel: 618314513
Martin Mateo, Concepcion	Fastenrath 65, 08035 Barcelona Tel: 934293197
Gimeno Dominguez, Carles	Judea 1-3, 08035 Tel: Barcelona Tel: 934184607
Penichet Guerra, Madeleine	Besós 27, 08035 Tel: Barcelona Tel: 933589601
Bosch De Basea Cusco, M. Dolors	Pantà De Tremp 63, 08032 Barcelona Tel: 934208035
Vilas Maso, Ana	Av. República Argentina 270, 08023 Barcelona Tel: 934173045
Mariné Serra, Carlos Enrique	Sant Eudald 63, 08023 Barcelona Tel: 932138476
Sánchez I Gea, Noelia	Mare De Deu Del Coll 99, 08023 Barcelona Tel: 932845126
Moreno Yuste, Maria	Gomis 82, 08023 Barcelona Tel: 934170810
Fuente Llobet, Joan	Mora d’Ebre 43, 08023 Barcelona Tel: 932138321
Roig Escobar, Berta	Pg. Mare De Deu Del Coll 150, 08023 Barcelona Tel: 932138210
Soler Ferran, Nuria	Av. De Vallcarca 125, 08023 Barcelona Tel: 934172320
Crespo Molina, Judith	Av. De Vallcarca 252, 08023 Barcelona Tel: 934180051
Leon Berrocal, Maria Elena	Farigola 48, 08023 Barcelona Tel: 932198773
Triginer Batalla, Rosa Ma	Pg. De La Vall D’Hebron 58, 08023 Barcelona Tel: 932115886
Rubio Gamarro, Esther	Beat Almató 20, 08023 Barcelona Tel: 934594686

**Figure 1:**
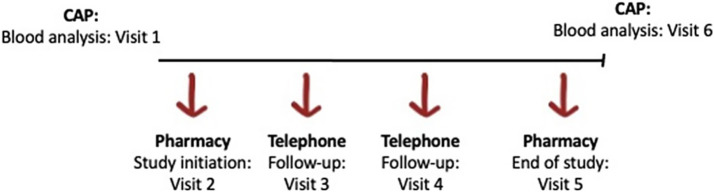
Design of patient 6 visits throughout the study.

The following study visits will be scheduled at the pharmacy chosen by each participant. A total of 4 visits at the chosen community pharmacy with the investigator pharmacist are expected for each participant (Figure 1).

An initial visit at the pharmacy will be performed (in person) with the participants (visit 2). During this visit, the investigator pharmacist from Blanquerna will obtain the signed informed consent, collect basic sociodemographic data (age, weight, height, and gender), and will perform the nutritional and well-being questionnaires and duration of current medication. Further, information about the personal trainer, the training schedule and the corporate Teams meeting links and password will be provided. During the first meeting, the participant will be provided with: the recommendations for a healthy diet by the Spanish Society of Diabetes and a nutritional proposal based on the abovementioned guidelines (33).

Two additional monthly check-ups, consisting of a short telephone interview (covering any adverse events, general well-being, etc.) will be performed 2 and 3 months after study initiation (visit 3 and visit 4).

A final visit at month 4 (visit 5), will be performed at the end of the study, in the pharmacy office where the well-being questionnaire and a satisfaction questionnaire will be provided. Upon completion of the study at the pharmacies, a second blood test at CAP Sant Rafael will be requested by the CAP physician; this procedure will take place during the 6th visit.

Pharmacists will oversee the follow-up, to ensure safety through rigorous monitoring and reporting procedures. Any concerning findings will be promptly addressed, and appropriate measures will be taken to safeguard participant health and ensure the integrity of the study results.

### 2.10 Safety

Measures to ensure the safety of participants include a thorough medical screening prior to enrollment, and a close monitoring of patients’ health status during intervention. Safety protocols also include the role of participating healthcare centers in case of any medical concerns or emergencies during the trial.

All adverse events, including hypoglycemia, hyperglycemia, musculoskeletal injuries, fatigue, and any other exercise-related symptoms, will be systematically collected. Adverse events will be assessed at each training session, at each pharmacy follow-up visit, during monthly telephone interviews, and at the final visit. The type, severity, and potential relation to the intervention will be documented. Serious adverse events will be reported immediately to the study’s principal investigators and the Ethics Committee. No interim efficacy analysis is planned due to the exploratory nature of this trial; however, the study will be stopped if ≥20% of participants in any arm experience serious adverse events related to the intervention.

In addition, participants will be provided with information on participating community pharmacies to receive appropriate assistance if needed. Additionally, participants will be educated on signs and symptoms of hypo-and hyperglycemia and instructed on appropriate actions to take if these occur during or after exercise sessions. The trial protocol also includes provisions for modifying or discontinuing exercise interventions based on individual participant responses and safety concerns identified during the trial.

Physical activity is not expected to produce adverse events. Nonetheless, physical activity programs will be individualized based on the needs and characteristics of each participant. If any adverse events related to physical activity are detected, patients can go to healthcare centers following standard practice.

### 2.11 Statistical analysis

All data will be reported as mean and standard deviation. Data normality and homogeneity of variance will be tested using Shapiro–Wilk and Levens tests, respectively. The primary endpoint (change in HOMA-IR) from baseline to 16 weeks, will be analyzed using ANCOVA with group as the fixed factor, baseline HOMA-IR as covariate, and VitD treatment status included as a stratification variable. Secondary continuous outcomes (glycemic, lipid, inflammatory, anthropometric, dietary, and psychological well-being measures) will also be analyzed with ANCOVA, adjusting for baseline values. Categorical outcomes (e.g., adverse events) will be compared using χ^2^ or Fisher’s exact test. Bonferroni *post hoc* test will be used if there is a significant difference between the groups. Exploratory analyses will test for interaction effects between exercise and VitD status using factorial ANOVA models; these analyses are considered hypothesis-generating due to sample size limitations. In addition, exploratory analyses will be performed to examine potential sex-related differences in primary and secondary outcomes. Sex will also be included as a covariate in the statistical models when appropriate. Statistical power will be calculated by *post hoc* power analysis method by using G Power software.

The results will be analyzed using SPSS (version 22) for Windows. The significant level (*α*) of all statistical analyses will be 0.05.

## 3 Discussion

The DIAVITEX study addresses a relevant clinical question of two widely recommended interventions for T2D: physical exercise and VitD supplementation. While both have shown independent benefits on insulin resistance, glycemic control, and cardiovascular health, few studies have evaluated their combined effects in a rigorously controlled, randomized clinical trial. This study is among the first to investigate their potential synergistic impact in a well-characterized cohort of T2D patients under real-world treatment regimens.

Previous literature has demonstrated that structured aerobic and resistance training significantly improve cardiorespiratory fitness and reduce HbA1c levels in patients with T2D (6). Similarly, low serum VitD levels are associated with impaired glucose metabolism and increased T2D risk (16). However, evidence regarding the effects of VitD supplementation alone has been inconsistent, particularly in individuals with normal baseline VitD status. Notably, few clinical trials have jointly evaluated these two strategies, and those that have done so often suffer from small sample sizes, limited outcome measures, and short intervention durations (20, 21).

DIAVITEX contributes to this knowledge gap by employing a factorial design that enables the comparison of exercise-only, VitD-only, combined, and control groups. By incorporating a broad range of clinical endpoints (including metabolic markers, inflammatory biomarkers, body composition, psychological well-being, and dietary adherence) the study offers a holistic perspective on the intervention effects. Additionally, the integration of pharmacists and primary care professionals reflects a translational approach that could facilitate future implementation in community settings.

An innovative aspect of this study is the delivery of the exercise program in a home-based, online format, which enhances accessibility and scalability. While this design reduces logistical barriers, it also introduces variability in adherence and exercise quality, which will be monitored, as discussed in the limitations. Importantly, all participants will receive standardized dietary advice to reduce confounding effects from uncontrolled nutritional factors.

The findings of this study have the potential to inform evidence-based clinical guidelines and public health policies by identifying whether combining exercise with VitD supplementation provides additive or synergistic benefits beyond either intervention alone. If successful, DIAVITEX could help justify broader recommendations for personalized, integrative care models in T2D management.

### 3.1 Limitations

Some potential limitations have been identified. One of them is related to the presence of T2D patients on metformin and iSGLT2 and already taking VitD. However, a pre-screening at CAP Sant Rafael has identified enough participants. Additional CAPs have been identified if more patients are needed, and physicians are willing to participate if required. Another potential limitation is related to exercise intervention. Exercise sessions are conducted online, which increases accessibility but prevents real-time supervision, potentially affecting adherence and proper technique. Several strategies will be applied to strengthen intervention fidelity, including the realization of an individualized familiarization session provided before the intervention, real-time online supervision, weekly pharmacy follow-up, monthly telephone interviews, and systematic review of exercise logs. Because the 1RM is estimated online rather than directly measured in person, accuracy may be reduced. However, the use of validated submaximal equations, real-time trainer supervision, perceived exertion monitoring, and follow-up is expected to mitigate this limitation. The 16-week intervention may not be sufficient to observe long-term metabolic changes or sustained behavioral effects. VitD levels are influenced by seasonal sun exposure, which may confound results, especially in participants not receiving supplementation. Nonetheless, recruitment and follow-up are done together during the same seasons, and serum VitD will be measured at both baseline and endpoint to account for these fluctuations. Also, due to practical reasons, the possibility to blind is restricted, which might be seen as a limitation.

Although validated tools (MEDAS and FFQ) will be used, self-reported dietary data remain subject to reporting bias. For this, complementary checks through follow-up interviews and optional dietary logs are incorporated to mitigate this limitation.
